# Exploring the role of the posterior middle temporal gyrus in semantic cognition: Integration of anterior temporal lobe with executive processes

**DOI:** 10.1016/j.neuroimage.2016.05.051

**Published:** 2016-08-15

**Authors:** James Davey, Hannah E. Thompson, Glyn Hallam, Theodoros Karapanagiotidis, Charlotte Murphy, Irene De Caso, Katya Krieger-Redwood, Boris C. Bernhardt, Jonathan Smallwood, Elizabeth Jefferies

**Affiliations:** a Department of Psychology and York Neuroimaging Centre, University of York, UK; b McConnell Brain Imaging Centre, Montreal Neurological Institute and Hospital, McGill University, Montreal, QC H3A 2B4, Canada

**Keywords:** Semantic control, Memory retrieval, Default mode network, Multidemand network, Posterior middle temporal gyrus

## Abstract

Making sense of the world around us depends upon selectively retrieving information relevant to our current goal or context. However, it is unclear whether selective semantic retrieval relies exclusively on general control mechanisms recruited in demanding non-semantic tasks, or instead on systems specialised for the control of meaning. One hypothesis is that the left posterior middle temporal gyrus (pMTG) is important in the controlled retrieval of semantic (not non-semantic) information; however this view remains controversial since a parallel literature links this site to event and relational semantics. In a functional neuroimaging study, we demonstrated that an area of pMTG implicated in semantic control by a recent meta-analysis was activated in a conjunction of (i) semantic association over size judgements and (ii) action over colour feature matching. Under these circumstances the same region showed functional coupling with the inferior frontal gyrus — another crucial site for semantic control. Structural and functional connectivity analyses demonstrated that this site is at the nexus of networks recruited in automatic semantic processing (the default mode network) and executively demanding tasks (the multiple-demand network). Moreover, in both task and task-free contexts, pMTG exhibited functional properties that were more similar to ventral parts of inferior frontal cortex, implicated in controlled semantic retrieval, than more dorsal inferior frontal sulcus, implicated in domain-general control. Finally, the pMTG region was functionally correlated at rest with other regions implicated in control-demanding semantic tasks, including inferior frontal gyrus and intraparietal sulcus. We suggest that pMTG may play a crucial role within a large-scale network that allows the integration of automatic retrieval in the default mode network with executively-demanding goal-oriented cognition, and that this could support our ability to understand actions and non-dominant semantic associations, allowing semantic retrieval to be ‘shaped’ to suit a task or context.

## Highlights

• Posterior middle temporal gyrus supports semantic control and event semantics.
• pMTG is at nexus of the default mode and multiple-demand networks.
• It links anterior temporal and prefrontal regions for representation and control.
• pMTG connects with inferior frontal gyrus during tasks and at rest.
• These sites form a semantic control network distinct from executive control.

## Graphical abstract

**Figure:**
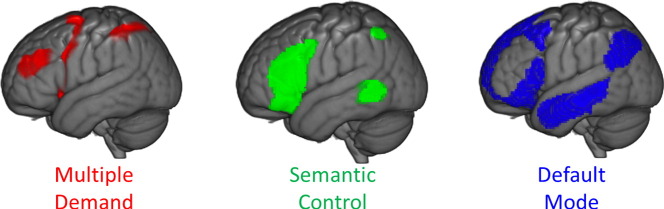

## Introduction

Across our lifetime we acquire a large body of conceptual knowledge, only a subset of which is relevant for any given task or context; thus automatic spreading activation within semantic representations is often insufficient for efficient semantic cognition (Thompson-Schill et al., 1997, Badre et al., 2005, Jefferies, 2013). Automatic spreading activation can facilitate the retrieval of features and associations that are *dominant* for a particular concept (e.g., carrot-peel). When semantic retrieval needs to be focussed on aspects of knowledge that are *not* the strongest response for the inputs, additional control mechanisms can be engaged to guide semantic retrieval. For example, control is needed to recover weak associations (carrot-horse) and to match words on the basis of specific sensory-motor features, such as actions or colour (e.g., carrot with traffic cone), since the functional characteristics of these concepts are more central to their meaning (Thompson-Schill et al., 1997, Badre et al., 2005, Whitney et al., 2011, Noonan et al., 2013, Davey et al., 2015a).

Different brain regions have been implicated in the *representation* and *controlled retrieval* of semantic information. The ventral anterior temporal lobes (ATLs) have been argued to form a key repository of conceptual information, following studies of patients with semantic dementia (SD). These patients have relatively focal bilateral atrophy focussed on ATL, associated with a gradual deterioration of knowledge and multimodal semantic deficits, first affecting fine-grained distinctions between concepts, and then eroding more basic distinctions (Mummery et al., 2000, Hodges and Patterson, 2007, Patterson et al., 2007). Deficits in SD patients suggest they show a loss of central semantic information (Bozeat et al., 2000, Jefferies and Lambon Ralph, 2006) and studies employing inhibitory transcranial magnetic stimulation (TMS) in healthy participants have provided converging evidence for a necessary role of this region in comprehension (Pobric et al., 2007, Pobric et al., 2010). Functional magnetic resonance imaging (fMRI) studies reveal activation of ATL during diverse semantic judgements (Binder et al., 2009, Visser et al., 2010, Rice et al., 2015). Finally, analyses of inter-regional signal correlations during task free (i.e. resting-state) functional scans have shown that ATL is part of a large scale assembly that includes medial prefrontal and posterior cingulate cortices, commonly referred to as the *default mode network* (DMN, Raichle et al., 2001, Buckner et al., 2008, Yeo et al., 2011, Jackson et al., 2016).

Converging neuroscientific methods have also identified brain regions beyond ATL which are important for multimodal semantics, specifically left inferior frontal gyrus (LIFG) and posterior middle temporal gyrus (pMTG). These regions are thought to contribute to the *control* of semantic retrieval. Patients with semantic aphasia (SA), who have lesions affecting these regions following stroke, fail the same range of verbal and non-verbal semantic tasks as SD patients; however, unlike SD cases, they often retrieve information that is irrelevant or inappropriate for the task, show strong effects of cues and miscues, and perform poorly in the face of strong distracters or ambiguous meanings (Thompson-Schill et al., 2002, Jefferies and Lambon Ralph, 2006, Jefferies et al., 2008, Jefferies et al., 2010, Corbett et al., 2009). Converging evidence from fMRI (Poldrack et al., 1999, Badre et al., 2005, Snijders et al., 2010, Noonan et al., 2013, Davey et al., 2015b) and TMS (Hoffman et al., 2010, Whitney et al., 2011, Davey et al., 2015a) supports the view that both of these regions contribute to semantic control. Indeed, in a recent neuroimaging meta-analysis, LIFG and pMTG were the sites activated most strongly and consistently across many different contrasts designed to tap semantic control (Noonan et al., 2013). In addition, when high-control semantic tasks were contrasted with demanding phonological judgements, pMTG and the anterior part of LIFG showed a specifically semantic response, suggesting that these two regions lie outside of the multiple-demand network (MDN), which is recruited during executively-demanding tasks across domains (Duncan, 2010).

These findings therefore provide some evidence that semantic cognition may be underpinned by at least three component processes, supported by distinct brain networks. (1) Domain-general executive control implemented by the MDN (Duncan, 2010) and the fronto-parietal control system (Power and Petersen, 2013) may support the capacity to engage and sustain a particular type of semantic retrieval in line with the task instructions, as well as the application of top-down constraints to support goal-driven aspects of cognition beyond semantics (Duncan and Owen, 2000, Duncan, 2010, Fedorenko et al., 2013, Noonan et al., 2013). For example, in a feature-matching task (in which globally unrelated words must be linked together on the basis that they both have a particular feature specified in the task instructions), there is a need to apply a pre-specified goal during semantic retrieval, and the implementation of this goal may involve the executive system. (2) Activation is thought to spread automatically between highly-related concepts within the representational system (underpinning semantic priming effects for strong associates). This allows dominant features and associations to be retrieved in the absence of executive control, and is supported by ATL and potentially other regions in the DMN (Wirth et al., 2011, Lau et al., 2013, Power and Petersen, 2013, Jackson et al., 2016). (3) A third network might support situations in which there is *no explicit goal* to indicate which aspect of knowledge should be brought to the fore, but the pattern of retrieval that is required for success is not the dominant one given the stimuli — i.e., semantic retrieval must be controlled to identify and sustain a linking context. The retrieval of relatively weak global associations is a good example of such a task: here, the instructions do not establish which types of associations or features should be the focus for retrieval — instead, it is necessary to establish a linking context from the concepts themselves and retrieve features relevant to this context.

**Fig. 1:**
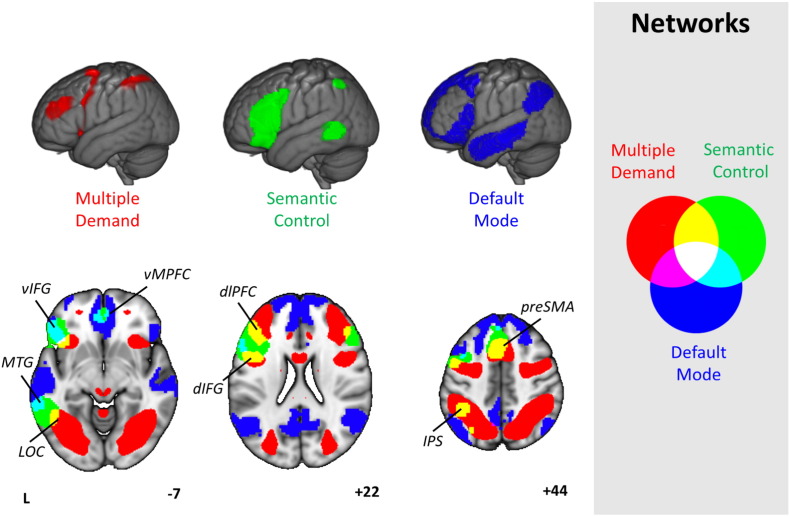
Spatial maps of the Default Mode Network (DMN, blue, from Yeo et al., 2011), Multiple-Demand Network (MDN, red, from Fedorenko et al., 2013) and Semantic Control Network (green, from Noonan et al., 2013), presented on a rendered MNI-152 brain and on axial, coronal, and sagittal slices. The key for overlapping areas between different networks is presented on the right hand side of the figure. Images are shown with fully saturated colours to maximise the visibility of the overlapping regions. Regions implicated in semantic control and also found in the MDN include dlPFC (dorsolateral prefrontal cortex), dIFG (dorsal inferior frontal gyrus), pre-SMA (pre-supplementary motor area), IPS (intraparietal sulcus) and LOC (lateral occipital cortex). Regions implicated in semantic control and also found in the DMN include vIFG (ventral inferior frontal gyrus); vMPFC (ventral medial prefrontal cortex) and pMTG (posterior middle temporal gyrus).

Fig. 1 illustrates the spatial distribution for these three putative networks (MDN, DMN, and semantic control) from prior published investigations. This figure shows that regions implicated in semantic control by the meta-analysis of Noonan et al. (2013, in green) are only partially overlapping with the MDN (from Fedorenko et al., 2013, in red). Non-overlapping areas in LIFG and pMTG appear to be important for demanding semantic tasks (relative to easier semantic judgements) but not executive control across domains. Moreover, these semantic control regions are spatially intermediate between the MDN (implicated in executive control) and the DMN (implicated in automatic retrieval, from Yeo et al., 2011, in blue); this location could allow semantic control regions to integrate two distributed networks that are anti-correlated at rest and yet both crucial for semantic cognition, e.g., when semantic knowledge, not a task goal, defines the attentional focus.

The proposal that the control of semantic retrieval is partially distinct from executive control is broadly consistent with functional dissociations that have been identified within left inferior frontal cortex. Within the language domain, studies have reported a functional gradient in left inferior frontal gyrus (IFG), with ventral anterior aspects of IFG implicated in semantic control specifically, and dorsal posterior IFG contributing more broadly to language control, including phonological tasks (Poldrack et al., 1999, Wagner et al., 2001a, Wagner et al., 2001b, Devlin et al., 2003, Gough et al., 2005, Snyder et al., 2007). Dorsal IFG, bordering inferior frontal sulcus (IFS), is recruited when participants select specific aspects of knowledge in line with an externally-specified goal (i.e., instructions to match words by colour or shape in the absence of a global semantic relationship; Badre et al., 2005). This selection process may be important for many language tasks, such as lexical and phonological retrieval. In contrast, ventral/anterior IFG shows an increased response when weak and strong semantic associations are contrasted (e.g., salt-grain > salt-pepper) — i.e., when participants shape retrieval to converge on a distant link between two concepts in the absence of an explicit goal. This ability to recover a non-dominant conceptual link does not generalise easily to other aspects of language processing. Recent work using single-subject analyses identified regions within the multiple-demand network, in dorsal and posterior IFG/IFS, that respond to difficult verbal working memory judgements involving non-words (Fedorenko et al., 2013): these regions are adjacent to, but spatially distinct from, areas of IFG that show a greater response to easier meaning-based trials involving words in sentences (Fedorenko et al., 2012, Blank et al., 2014). Moreover, analyses of resting-state connectivity have implicated anterior aspects of prefrontal cortex in a cingulo-opercular control system, which includes regions that display sustained activity during task-set maintenance, while dorsal prefrontal regions couple with a fronto-parietal system engaged by ongoing selection and implementation (Power and Petersen, 2013): this pattern may relate to the functional distinction between anterior and dorsal LIFG. Thus, a more semantic response in anterior/ventral parts of IFG may be broadly in line with the proposal that anterior areas in IFG establish and maintain priorities for what is to be retrieved, while the short-term process of selection itself is implemented in posterior regions of IFG (Badre and D'esposito, 2007). Badre and colleagues referred to this functional specialisation within IFG as “controlled retrieval” and “selection” respectively (Badre et al., 2005).

The functional contribution of IFG has been considered in detail while the significance of the second region identified by Noonan and colleagues, pMTG, remains controversial. Although this site is implicated in semantic control, a parallel literature links pMTG, together with angular gyrus (AG), to the comprehension of actions and events (Johnson-Frey et al., 2005, Liljeström et al., 2008), and to relational semantics (Humphreys and Lambon Ralph, 2014, Price et al., 2015), and these adjacent areas of temporoparietal cortex can show a similar response to contrasts tapping event knowledge (Wagner et al., 2005, Sachs et al., 2008, Kim, 2011). One theoretical account suggests that AG and/or pMTG provide a “thematic hub”, capturing aspects of knowledge relating to the associations between concepts — such as knowledge about which concepts are found and used together (Schwartz et al., 2011). However, while pMTG often shows increased activation in harder semantic tasks (Noonan et al., 2013), mid-AG typically shows deactivation in semantic and other tasks relative to rest, especially for harder judgements (Binder et al., 2008, Humphreys and Lambon Ralph, 2014, Humphreys et al., 2015). Moreover, these sites showed a double dissociation in a recent TMS study (Davey et al., 2015a): inhibitory stimulation of pMTG disrupted weak more than strong associations, while TMS to AG showed the opposite pattern. These data are not easily reconciled with a simple “thematic hub” account and suggest instead that pMTG and AG support different components of semantic cognition, although ones that can at times function in a cooperative manner.

The current study focussed on understanding the functional contribution of pMTG to semantic control and event/relational semantics: we explored the hypothesis that this region acts to integrate information from the MDN and also the DMN, which are anti-correlated at rest. First, we identified whether there are regions of pMTG that show a *common* response to the retrieval of action features (contrasted with colour feature judgements which have not been historically linked to pMTG; Badre et al., 2005) and global associations (relative to feature judgements). Both of these event/relational contrasts depend on retrieving information in line with a specific stimulus-driven context, as opposed to the application of a specific goal specified in the instructions. We compared the location of this response to regions implicated in semantic control and domain-general control in previous meta-analyses and also used psychophysiological interaction (PPI) analysis to understand the functional coupling of this region under these conditions. Second, to explore whether pMTG could *integrate* executive and automatic mechanisms contributing to semantic retrieval, we used resting state fMRI and diffusion MRI tractography to examine if the spatial networks that corresponded to peaks in our easy and hard semantic decisions (i.e., the contrasts of relatively global associations over the harder feature selection and vice versa), converged on the region of cortex identified as important in event/relational semantics. Third, we considered whether the response in pMTG could be linked to the proposed functional gradient in IFG (Badre et al., 2005) by examining whether ventral/anterior as opposed to dorsal/posterior IFG had greater resting state connectivity with this region.

## Method

### Participants

This research was approved by the ethics committee of the York Neuroimaging Centre, University of York, UK. All participants were right-handed, native English speaking, with normal or corrected-to-normal vision. For the experimental task, we recruited 22 neurologically healthy participants from the University of York (Cohort 1). Two participants were removed due to movement artefacts during fMRI data acquisition (mean age = 24.8, SD = 3.8, range 21–35 years, 9 males). Resting-state scans were collected at York for two cohorts (mean age = 21.3, SD = 2.7, range 18–31 years, 38 males); 39 participants were recruited into Cohort 2, with 48 recruited into Cohort 3. These two samples were collected as part of different projects; however the resting-state scan was collected before task-based scans in both cases and the data sets are combined in the analysis below. For Cohort 3, we also obtained diffusion MRI. Finally, we also used two publicly-available data sets to provide independent confirmation of the resting-state connectivity patterns observed in this study: (i) 141 participants from the Nathan Kline Institute (NKI)/Rockland Enhanced Sample (Nooner et al., 2012) were used to relate the connectivity patterns of ventral/anterior and dorsal/posterior IFG to the response we observed in pMTG. Full details of this sample can be found in Gorgolewski et al. (2014). (ii) Data from Yeo et al. (2011), implemented in Neurosynth (Yarkoni, 2011), was used in a final step to investigate the functional connectivity of the pMTG site we obtained across different analyses. We compared the functional behaviour of the pMTG across these different cohorts to minimise the duration of specific testing sessions and to allow us to capitalize on the power of large scale publicly available data sets.

### Study design

**Fig. 2:**
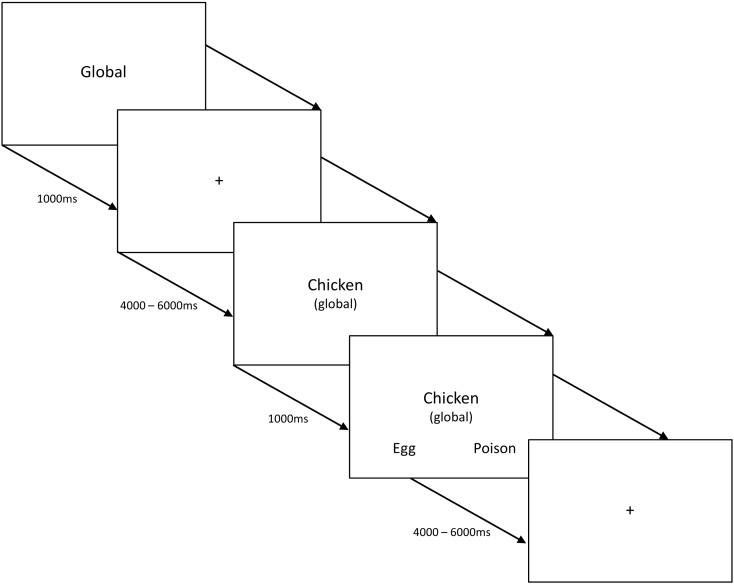
Example trial structure for all conditions in the task-based fMRI study. The study employed a 2 × 3 design, with three types of judgements (about global semantic associations, size feature matching and specific feature matching) for animal and tool concepts.

Semantic knowledge for items from two semantic categories (animals and tools) was probed using three tasks (see Fig. 2). (*i*) The first task *(global associations*) involved matching probe words to a semantically-related target (e.g., selecting honeycomb for the probe bee, as opposed to an unrelated distracter). This task did not require participants to apply a specific goal or instruction to constrain semantic retrieval; instead it was necessary to identify a semantic link from the items presented on each trial. (*ii*) In the second task, participants were asked to identify a target which had similar dimensions to the probe concept (*size matching*) (e.g., selecting flannel for the probe sandpaper, as these items are similar in size/shape, even though they are not globally related). (*iii*) The final tasks required participants to match items based on highly specific features (*specific feature matching*). For tool items, participants were asked to select the target word that had a similar action to the probe word (e.g., selecting screwdriver for the probe key, as these tools involve similar turning actions). For animals, participants matched items on the basis of colour similarity (e.g., selecting basketball for the probe tiger, as both are orange and black). Both tasks *ii* and *iii* required participants to match items based on a feature given to them in the task instructions, as opposed to identifying a link from the stimuli themselves. However, the specific feature matching tasks, based on colour and action, were harder (see behavioural data below). Given our research question, we focussed on the following contrasts: first, the conjunction of action > colour features (localising voxels that respond to action understanding) and global associations > size features (localising voxels that respond to relational judgements), expected to converge in pMTG. By looking at the conjunction of these two sets of contrasts, we can rule out task difficulty as a confound in our localisation of the pMTG (since the global association task was easier than size matching). Secondly, we contrasted the hardest feature selection tasks (action and colour matching) with easier global associations, to identify brain regions responding differentially to control demands (and the reverse contrast for more automatic spreading activation).

The experiment was organised into a total of 36 blocks divided equally among the 6 experimental conditions (i.e., the 3 tasks probed using 2 categories). There were 5 trials per block resulting in 30 trials per experimental condition. Before each block commenced, an instruction slide was presented stating the task to be performed (global, size, action, or colour) for 1000 ms. A reminder of the instructions was also present on each trial in parentheses under the probe word. A two-alternative forced choice paradigm (Fig. 2) was used; participants were instructed to match the centrally presented probe word to one of two potential targets. Probe words were presented for 1000 ms, followed by the response options which remained on screen until a response was recorded via button press, with maximum trial duration set to 4500 ms. The inter-trial interval was 4000–6000 ms, with 10 s of rest between each experimental block. One null event was present in each experimental block to increase the amount of rest which was used as a baseline in the analysis of the fMRI data; the screen was blank for 4500 ms plus jitter (4000–6000 ms) with the location of the null event randomised in each experimental block. Before the fMRI experiment, participants were given a practice session consisting of two blocks for each condition. The task was presented in the scanner using NBS Presentation version 16. Participants viewed the words via a front silvered mirror and responded using a Lumina Response Pad (Cedrus Corporation), placed in their left hand.

### Stimuli

A copy of the stimuli used in this experiment is provided using the Open Science Framework (OSF, https://osf.io/); https://osf.io/5pq8z/. All words used in the experiment were concrete nouns denoting manipulable objects or animals. There were 30 animal probes and 30 man-made probes, repeated across the three tasks (global association, size matching and specific-feature matching), each with a unique target word for each task. The distracters in all conditions were target items from other trials that did not have overlapping features or a global association with the probe. No restrictions were placed on the number of times a word could be used (mean number of repetitions = 2, SD = 1.4, range = 6); however the number of repetitions was equivalent between conditions. For the words in each trial (probe, target, and distracter) we collected measures of familiarity, imageability, manipulability, lexical frequency, word length, and number of words, averaging across all the words in a single trial, and compared trials across relevant conditions. Ratings of familiarity and imageability were taken from the MRC psycholinguistic database (Wilson, 1987). Ratings of manipulability, familiarity, and imageability were also collected on a 7 point scale (1 — low, 7 — high) from a separate cohort of 11 healthy adult participants who did not take part in the scanning sessions (familiarity and imageability ratings were collected for targets with missing values in existing databases). Lexical frequency was taken from the SUBLEX-UK database (van Heuven et al., 2014). Table 1 contains the psycholinguistic variables for the experimental conditions. Trials were matched across conditions for word length (action vs. colour: *t*(58) = 1.1, p = .276; global vs. size:, *t*(58) = 1.89, p = .076; global vs. feature: *t*(58) = 1.46, p = .179). They were also matched for number of letters (action vs. colour: *t*(58) = .14, p = .886; global vs. size: *t*(58) = .23, p = .818; global vs. feature: *t*(58) = .75, p = .449), and lexical frequency (action vs. colour: *t*(58) = 1.48, p = .114; global vs. size: *t*(58) = 1.13, p = .261; global vs. feature: *t*(58) = 1.43, p = .156). Manipulability ratings were higher for action than colour trials as expected (*t*(58) = 9.47, p < .001). Imageability was also higher for colour than action trials (*t*(58) = 7.61, p < .001). No significant differences were observed for manipulability and imageability for global vs. size (manipulability: *t*(58) = .027, p = .979; imageability: *t*(58) = 1.55, p = .123), or for global vs. feature (manipulability: *t*(58) = .50, p = .616; imageability: *t*(58) = .322, p = .748). Finally, a different set of 13 participants rated the extent to which they found it necessary to generate a spatiotemporal context to complete the specific feature matching conditions (e.g., colour and action judgements), on a seven point scale (1 — not very useful, 7 — retrieving the context was very helpful). Retrieval of a spatiotemporal context was significantly more important for tool action trials (M = 4.13, SD = .50) than animal colour trials (M = 2.49, SD = .43; *t*(28) = 13.87 p < .001).

### MRI acquisition

Structural and functional data were acquired using a 3T GE HDx Excite MRI scanner utilising an eight-channel phased array head coil (GE) tuned to 127.4 MHz, at the York Neuroimaging Centre, University of York. Structural MRI acquisition in all participants was based on a T1-weighted 3D fast spoiled gradient echo sequence (TR = 7.8 ms, TE = minimum full, flip angle 20°, matrix size = 256 × 256, 176 slices, voxel size = 1.13 × 1.13 × 1 mm^3^). Task-based and resting-state activity was recorded from the whole brain using single-shot 2D gradient-echo echo planar imaging (EPI) with a flip angle = 90°, matrix size = 64 × 64, and field of view (FOV) = 192 × 192 mm^2^. Other scan parameters slightly varied for task-based fMRI in Cohort 1 (TR = 2000 ms, TE = 30 ms, 32 slices, voxel size = 3 × 3 × 4.5 mm ^3^, 12 min), resting-state fMRI for Cohort 2 (TR = 2000 ms, TE = minimum full, 32 slices with 0.5 mm gap, voxel size = 3 × 3 × 3 mm^3^, 7 min) and resting-state fMRI for Cohort 3 (TR = 3000 ms, TE = minimum full, 60 slices, voxel size = 3 × 3 × 3 mm^3^, 9 min). An intermediary FLAIR scan with the same orientation as the functional scans was collected to improve the co-registration between subject-specific structural and functional scans. In Cohort 3, we also collected diffusion weighted MRI data using a 2D single-shot pulsed gradient spin-echo EPI sequence (TR = 15,000 ms, TE = 86 ms, matrix = 96 × 96, 59 slices, voxel size = 2 × 2 × 2 mm^3^; b = 1000 s/mm^2^, 45 diffusion directions, 7 B0 volumes, 13 min). Parameters of the independent (NKI)/Rockland Enhanced Sample are described in detail by Gorgolewski et al. (2014) and Smallwood et al. (2016).

### Data pre-processing and analysis

#### Task-based fMRI

Analyses were conducted at the first and higher level using FSL-FEAT version 4.1.9 (Smith et al., 2004, Woolrich et al., 2009, Jenkinson et al., 2012). Pre-processing included slice timing correction, linear motion correction (Jenkinson et al., 2002), high-pass temporal filtering (sigma = 100 s), brain extraction (Smith, 2002), linear co-registration to the corresponding T1-weighted image followed by linear co-registration to MNI152 standard space (Jenkinson and Smith, 2001), spatial smoothing using a Gaussian kernel with full-width-half-maximum (FWHM) of 5 mm and grand-mean intensity normalisation of the entire 4D data set by a single multiplicative factor.

Pre-processed time series data were modelled using a general linear model correcting for local autocorrelation (Woolrich et al., 2001) using a block design. The linear model included the six experimental conditions modelling block start time and block duration. fMRI scanning was split into two separate scanner runs collected sequentially; both runs were analysed independently at the lower level then combined using a fixed-effects higher-level analysis. Six contrasts were defined; individual conditions > rest (animal/tool global, animal/tool size, tool action, animal colour). We focussed our subsequent analysis on the comparison of easy global associations vs. harder specific feature selection (building on the approach of Badre et al., 2005) and on a conjunction of action > colour and global associations > size to identify regions engaged by event/relational semantics (Nichols et al., 2005). All analyses were cluster corrected using a z-statistic threshold of 2.3 to define contiguous clusters. Multiple comparisons were controlled using Gaussian Random Field Theory at a threshold of p < .05.

#### Psychophysiological interaction (PPI)

A conjunction analysis of action > colour and global > size revealed an area of pMTG that responded to event semantics. We used this pMTG region as a mask and extracted the time-course (for each participant and each run) within this area to examine psychophysiological interactions (PPI; O'Reilly et al., 2012) between the pMTG and other brain regions involved in event semantics. The extracted time-course of pMTG and the interaction were included in a GLM model as explanatory variables (at the lower level, for each participant and each task individually, for each run). As with the functional analysis, the two runs were combined, and the results were submitted to a group level analysis, with the same cluster-forming threshold and significance level (*Z* = 2.3, p < .05). The contrasts included in this analysis were action > colour and global > size as before — and, as with the functional data, a formal conjunction of these contrasts was conducted.

#### Resting-state fMRI

Pre-processing steps were as for task fMRI, apart from the addition of Gaussian low pass temporal filtering, with sigma = 2.8 s, and spatial smoothing using a Gaussian kernel with full-width-half-maximum (FWHM) of 6 mm. We extracted the time series from 3 mm spheres placed at regions of interest (ROIs, see below) and used these as explanatory variables in connectivity analyses at the single subject level. In each analysis, we entered 11 nuisance regressors; the top five principal components extracted from white matter (WM) and cerebrospinal fluid (CSF) masks based on the CompCor method (Behzadi et al., 2007) and six head motion parameters. WM and CSF masks were generated from each individual's high resolution structural image (Zhang et al., 2001). No global signal regression was performed, following the method implemented in Murphy et al. (2009). At the group-level, analyses were carried out using FMRIB's Local Analysis of Mixed Effects (FLAME1), the same cluster correction method used for the functional fMRI was used at the group level.

#### Diffusion MRI

Subject-wise diffusion MRI processing was carried out in native diffusion space using FSL (version 4.1.9). Pre-processing of the DTI data involved eddy-current distortion correction and motion correction using FDT v2.0 (part of FSL), as well as brain extraction using BET. A probabilistic diffusion model was then fitted on the corrected data using BEDPOSTX: the Bayesian estimation of diffusion parameters obtained using sampling techniques toolbox (Behrens et al., 2003). BEDPOSTX uses Monte Carlo Markov chain sampling to generate parameters for probabilistic tractography. Up to 2 fibres were modelled per voxel using a burn-in of 1000 iterations before starting the sampling of diffusion parameters. Next, probabilistic tractography was performed to reconstruct fibres passing through our seed masks using PROBTRACKX. This technique repeatedly samples from the diffusion parameters calculated in BEDPOSTX to build a distribution of the likely tracts from each seed region. The seed masks were transformed from MNI standard space to diffusion space using nonlinear registration. 5000 sample tracts were generated per seed voxel. We used the standard parameters of a curvature threshold of 0.2 (corresponding to a minimum angle of approximately ± 80°), a step length of 0.5 mm and a maximum number of steps of 2000. No waypoint or termination masks were included. The resulting individual maps were transformed back to MNI standard space, thresholded at 0.02% of total samples sent from the mask and concatenated into a single 4D file. Nonparametric voxelwise statistical testing was performed using FSL Randomize with 25,000 permutations in order to get a group tractography map (Nichols and Holmes, 2002). The resulting maps were thresholded at p < 0.01, Family-Wise Error (FWE) corrected, using the Threshold-Free Cluster Enhancement (TFCE) technique (Smith and Nichols, 2009).

### Selection of seeds and ROIs

For the psychophysiological interaction (PPI) analysis we used the cluster generated by the conjunction of action > colour and global > size mask. For the resting state analysis we used two functional peaks from our task-based fMRI analyses (data from Cohort 1) as seeds in the analyses of resting state connectivity and diffusion MRI (data from Cohorts 2 and 3): one functional peak was linked to relatively automatic semantic retrieval (easy global associations > hard feature selection, in inferior ATL, MNI co-ordinates − 48 2 − 38) and one was linked to executive control (hard feature selection > easy global associations, in IFS, MNI co-ordinates − 42 28 16). In a further resting-state connectivity analysis (using the NKI data), we placed seeds at peaks responding to different aspects of semantic control taken from Badre et al. (2005), allowing us to link the response in pMTG to the previously-reported functional distinction between “selection” in dorsal/posterior IFG (MNI − 48 18 18) and “controlled retrieval” in ventral/anterior IFG (MNI − 51 27 3).

We also performed an ROI analysis of the task-based fMRI data using 8 mm spheres, focused on these ventral and dorsal LIFG peaks from Badre et al. (2005) and the pMTG peak for semantic control taken from the Noonan et al. (2013) meta-analysis (− 58 − 49 − 8). The FEATquery tool in FSL was used to extract average percentage signal change across all the voxels in each ROI for all six conditions across participants.

Following Simmons et al. (2011) we report the design choices that our study depends on. The sample size of 22 for the functional data (Cohort 1) was based on the assumption that approximately 20 participants with useable data would be necessary to provide a stable measurement of the semantic processes in question. We used samples of approximately 50 participants for the diffusion MRI and 90 participants for the resting state analysis (Cohorts 2 and 3) reflecting the data that was available; moreover, prior studies conducted in our laboratory that have successfully revealed positive results with samples that range from 40–90 participants (e.g. Smallwood et al., 2013, Smallwood et al., 2016). For the NKI data, we used the same participants as in a previous investigation (Gorgolewski et al., 2014), since the data was already available. We did not perform a formal power calculation for any of these decisions.

The participants in Cohorts 2 and 3 who provided resting state and diffusion MRI subsequently performed a behavioural battery of tasks in the laboratory. These measures were not directly related to the current experimental question and were not explored in the current study. The relationship between these measures and individual variation in cognitive performance is an ongoing focus in our laboratory (for examples in the public record see Baker et al., 2015, Konishi et al., 2015, Smallwood et al., 2016).

## Results

### Behavioural results

Behavioural performance (reaction time, accuracy and response efficiency) is shown in Table 2. A 2 (category; animals vs. tools) by 3 (task; global association, size feature, and specific feature) repeated-measures analysis of variance (ANOVA) was conducted on response efficiency, revealing no significant differences between item categories (F (1,29) = 1.33, p = .258), and a significant main effect of task (F (2,58) = 19.28, p < .001), demonstrating poorest performance in the specific feature condition (M = 2313), followed by the size feature (M = 2088), and global association (M = 1655) matching conditions. No significant interaction was observed (F (2,58) = .42, p = .662). This pattern of results justifies the comparison of specific feature matching vs. global associations as a way of identifying regions responding to difficult judgements (cf. Badre et al., 2005).

### Neuroimaging results

The unthresholded statistical maps can be found on Neurovault; http://neurovault.org/collections/WLSYBFYI/. This collection contains the uncorrected z-statistic maps for the 6 experimental conditions (animal/tool global, animal/tool size, animal colour, and tool action) contrasted against rest.

We identified the region of pMTG important for event/relational semantics through a conjunction of two contrasts that commonly involved generating a spatiotemporal or thematic context to identify a link between items: (i) Global semantic associations (i.e., whether chicken goes with egg) were compared with feature decisions about object size (i.e., whether a tortoise is the same size as a helmet), since global associations to both animals and tools require a linking context to be recovered, while size matching does not. (ii) Decisions about action features (i.e., whether the motion used by a key is similar to a screwdriver), were compared with decisions about colour features (i.e., whether a Tiger is the same colour as a Basketball), since tool action judgements involved generating a spatiotemporal framework to support retrieval, while animal colour judgements did not. While these contrasts are different in many ways, pMTG was expected to respond to both since it has been implicated in understanding actions and thematic associations. Importantly, a response to this conjunction cannot be explained in terms of global task difficulty — since the global association task was easier than the size judgement task, according to behavioural performance.

**Fig. 3:**
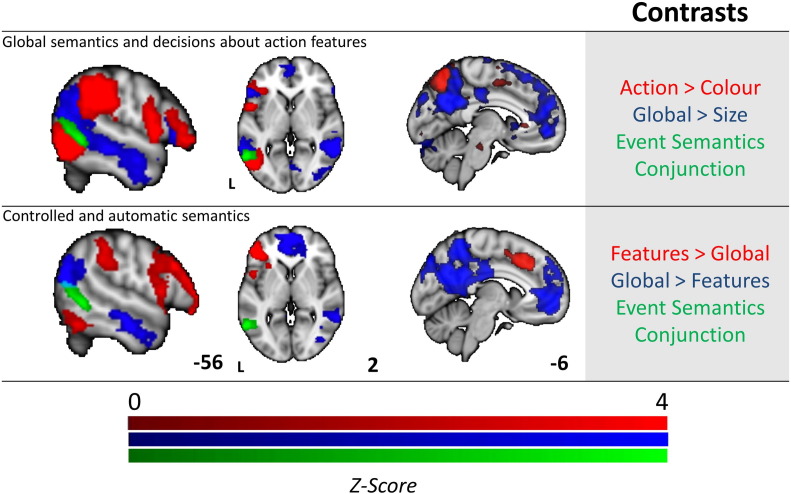
Functional activation for the conjunction of global semantic decisions and action features (top row), and the contrast between easy and hard semantic decisions (bottom row). The top row displays activation for global semantic associations > size feature selection (blue), and for tool action feature selection > animal colour feature selection (red). The conjunction revealing the shared activation between these contrasts (event/relational semantics) is shown in green. The bottom row shows the contrast of hard > easy semantic decisions (specific feature selection trials > easier global association judgements, in red) and easy > hard semantic decisions (the reverse contrast, in blue). The event semantics conjunction from the top row is overlaid (in green). All contrasts and conjunctions are cluster corrected for multiple comparisons (inclusion threshold z = 2.3, cluster significance = p < .05).

The result of these analyses is presented in Fig. 3. When compared to size judgements, global associations activated posterior aspects of the temporal lobes extending from the lateral occipital cortex, along the middle temporal gyrus into the anterior temporal lobes. Activation was also observed in the left frontal lobe focused on middle frontal gyrus, superior frontal gyrus and frontal pole (contrast shown in blue in top panel of Fig. 3). Relative to colour judgements, action judgements activated a large left temporoparietal cluster including inferior lateral occipital cortex, posterior middle and inferior temporal gyrus, supramarginal gyrus, superior parietal lobule and angular gyrus. A second inferior frontal cluster revealed activation in precentral gyrus, LIFG (opercularis and triangularis) and frontal orbital cortex (contrast shown in red in top panel of Fig. 3). The formal conjunction analysis between these contrasts revealed the hypothesised pattern of shared activation in pMTG, indicating that this region was common to both action and relational semantic judgements (shown in green in Fig. 3, both top and bottom panels).

To understand if this region of pMTG was independent of regions that were exclusively activated by either difficult or easy tasks we compared the conjunction in Fig. 3 (shown in green), with the contrasts of specific feature selection over global associations (hard > easy semantic decisions, shown in red in Fig. 3, bottom panel) and global semantic associations over specific feature selection (easy > hard semantic decisions, shown in blue in Fig. 3, bottom panel). Consistent with prior studies (Duncan and Owen, 2000, Fedorenko et al., 2013), hard > easy decisions engaged regions of the MDN, whereas easy > hard decisions activated regions in the DMN. The conjunction of action and relational judgements (in green in Fig. 3) fell between these networks suggesting that the pMTG's role in processing actions and relations is consistent with a possible role for this site in the integration of information from the multiple-demand network and DMN.

**Fig. 4:**
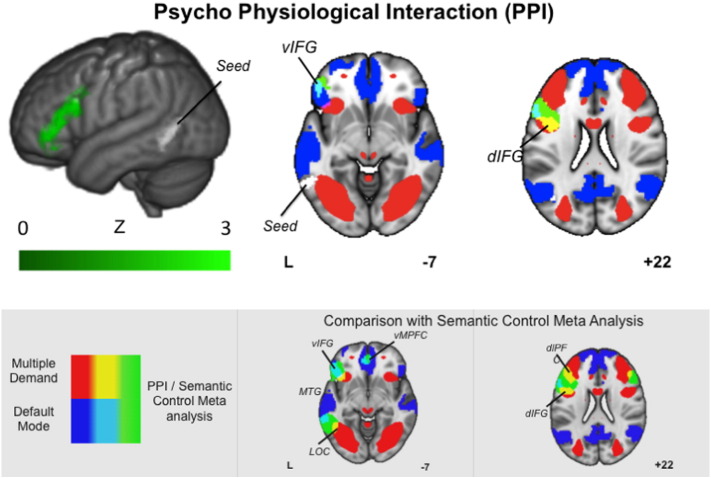
The rendered image shows the results of the psychophysiological interaction (PPI) analysis for the region showing common activation for the global semantic decisions and action features (green). The axial slices illustrate regions of overlap between this spatial map and the default mode network (DMN) and the multiple demand network (MDN). For ease of visual comparison the grey box presents the overlap between the meta-analysis of Semantic control and the same pair of large-scale networks. The PPI was cluster corrected for multiple comparisons (inclusion threshold z = 2.3, cluster significance = p < .05).

Next, to assess the possibility that the pMTG is part of a network of regions that is important for the functional integration of information from the MDN and the DMN, we conducted a psychophysiological interaction (PPI) analysis to characterise its functional connectivity during active task processing. We used the region of the pMTG that responded to the conjunction of Action > Colour and Global > Size as a seed region and at the group level explored the pattern of functional connectivity that was common to both forms of event/relational semantics. The results of this analysis are presented in Fig. 4: there was greater functional connectivity during event/relational semantics in a large region of inferior prefrontal cortex. Comparisons of this spatial map with the DMN revealed a region of overlap in the ventral inferior frontal gyrus, while a comparison with the MDN revealed overlap in more ventral regions of lateral prefrontal cortex.

**Fig. 5:**
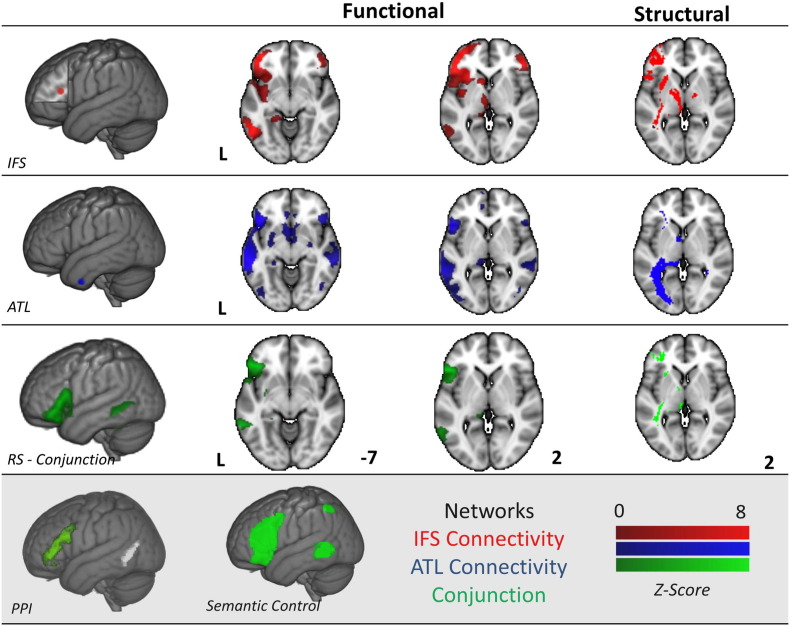
Resting state functional connectivity and structural connectivity (tractography) for functional peaks identified for hard semantic judgements in a key executive region (− 42 28 16, inferior frontal sulcus — IFS, in red) and easy semantic judgements in a region linked to semantic representation (− 48 2 − 38, anterior temporal lobe — ATL, in blue). The conjunction for the two connectivity patterns is displayed in green. All contrasts and conjunctions are cluster corrected to control for multiple comparisons (inclusion threshold z = 2.3, cluster significance = p < .05). The left-hand column shows the seed regions, columns 2 and 3 show resting state connectivity and white matter (WM) fibre tracts identified using diffusion MRI for each seed and their overlap are displayed on the right. We present the spatial maps from the Noonan et al. (2013) meta-analysis and from the prior PPI analysis to facilitate visual comparison of these three networks. Note the colour bar does not refer to the DTI Images which were corrected using randomise and are presented as fully saturated maps.

During tasks that rely on event/relational semantics, the pMTG shows increased activation and heightened communication with regions in the MDN and DMN, indicating a role for posterior regions of the temporal lobe in the integration between these two large-scale networks. To understand whether this integrative role arises because pMTG is at the nexus of the DMN and MDN we performed a seed based resting state fMRI connectivity analysis in an independent set of participants (Cohorts 2 and 3). We placed seeds around the peak response in IFS using the contrast of feature selection > global associations (to identify a region in the MDN implicated in executive control; − 42 28 16). We used the reverse contrast of global associations > feature selection in the ATL (to identify a site in the DMN implicated in semantic representation and automatic retrieval; − 48 2 − 38). A formal conjunction between these connectivity maps identified two regions common to both networks, in LIFG and pMTG (overlapping with the cluster found in the functional study; see Fig. 5). This spatial pattern was similar to the functional recruitment observed in the PPI analysis (see subpanel). In addition, we examined the fibre tracts from our ATL and IFS seed regions using diffusion tractography. Overlapping white matter (WM) tracts were observed beneath our conjunction pMTG cluster (see Fig. 5, right-hand column). These results suggest that the region of pMTG implicated in event/relational semantics, and in semantic control by the wider literature (Kim, 2011, Noonan et al., 2013, Davey et al., 2015b), has a pattern of functional and structural connectivity that would allow it to coordinate networks implicated in automatic semantic retrieval and executive control.

**Fig. 6:**
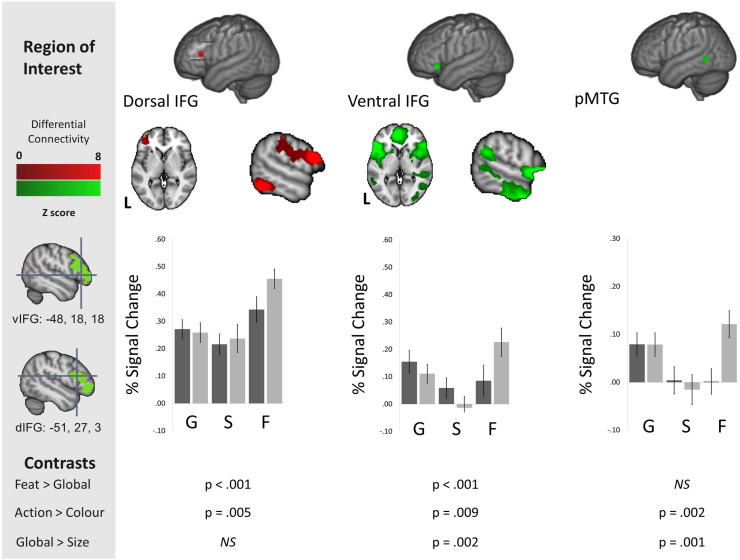
The upper panel shows the difference in resting-state connectivity between the dorsal (red) and ventral (green) inferior frontal gyrus (IFG) peaks from Badre et al. (2005), presented on MNI-152 axial and coronal slices. All difference maps are cluster corrected to control for multiple comparisons (inclusion threshold z = 2.3, cluster significance = p < .05). Colour bars represent the strength of the difference in the connectivity profiles between the two seed regions in LIFG. The lower panel shows the percent signal change for all experimental conditions extracted from 8 mm regions of interest (ROIs) for dorsal and ventral IFG from Badre et al. (2005) and for posterior middle temporal gyrus (pMTG) from Noonan et al. (2013). For ease of visual presentation we present the overlap between the seed regions in IFG and the resting state connectivity presented in Fig. 5 in the grey box. The black bars represent percentage signal change for the animal conditions, and the grey bars represent the signal change for the tool conditions. Error bars correspond to the standard error, with p values for between-condition t-tests presented below. G = global associations. S = size feature matching. F = specific feature matching.

We next related the region of pMTG implicated in the analyses above to functional gradients previously reported for control-demanding semantic tasks in LIFG. Our aim was to investigate whether this region shows a pattern of connectivity compatible with a large-scale distributed network underpinning a particular aspect of semantic control (i.e., Badre et al.'s, 2005 distinction between “controlled retrieval” in anterior/ventral IFG, and “selection” in posterior/dorsal IFG). We reasoned that if pMTG is implicated in specific aspects of controlled semantic retrieval (e.g., the generation and application of a spatiotemporal or thematic context to identify a link between concepts), as opposed to difficult semantic judgements in general, it may show stronger connectivity to anterior/ventral portions of IFG. Our prior PPI analysis yielded a cluster of activity that extended from the dorsal region corresponding to Badre et al.'s (2005) peaks for selection (− 48 18 18) to the ventral region corresponding to their peak for controlled retrieval (− 51 27 3) indicating that functionally the pMTG increased communicated with both of these regions during event semantics. To understand if the resting profile of the pMTG was more similar to the region important in controlled retrieval, we calculated the differences in the functional connectivity profiles of the two seeds using the NKI data set. Regions that showed greater connectivity with anterior/ventral IFG (compared with posterior/dorsal IFG) included anterior temporal lobes, medial prefrontal cortex, angular gyrus and a region of pMTG that overlapped with our previous task-based analysis (see Fig. 6 top panel). This illustrates that although during event/relational semantics, pMTG communicates with both dorsal and ventral regions of the inferior frontal cortex, at rest this region of posterior temporal cortex is more functionally coupled to ventral regions of inferior frontal cortex.

We also performed a region of interest (ROI) analysis of the task-based data to examine the correspondence between the pattern of response in anterior/ventral and posterior/dorsal IFG with the functional recruitment in pMTG, localised using the peak for semantic control from the meta-analysis of Noonan et al. (2013). This analysis revealed similarities between pMTG and anterior/ventral IFG — with both sites responding more to (i) global associations vs. size feature judgements, as well as (ii) action feature matching vs. colour feature matching (see Fig. 6 bottom panel). In contrast, posterior/dorsal IFG responded most strongly to the two specific feature selection tasks and showed no preference for the global association task over size matching: thus, percentage signal change in this dorsal region largely mirrored the difficulty of the conditions.

**Fig. 7:**
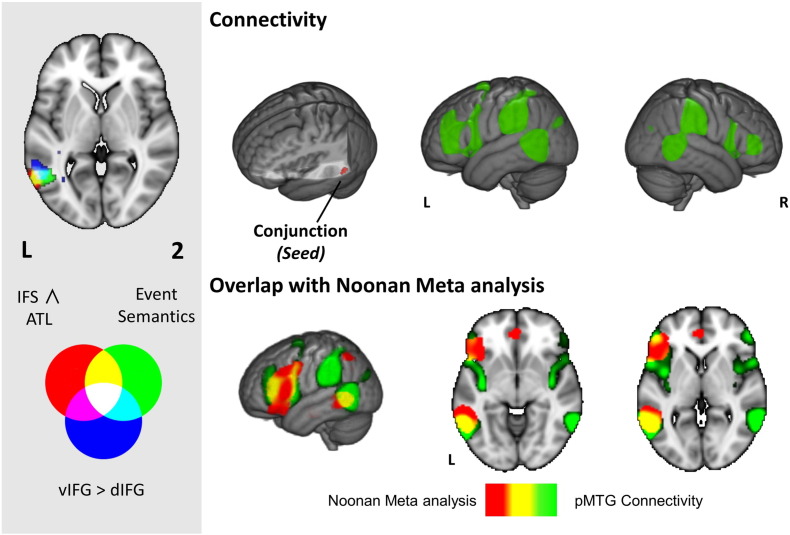
The left hand panel shows overlapping clusters in posterior middle temporal gyrus (pMTG) across different contrasts and analyses; (i) the event semantics task-based conjunction (green, reproduced from Fig. 3), (ii) the inferior frontal sulcus (IFS) and anterior temporal lobe (ATL) connectivity conjunction (red, reproduced from Fig. 5), and (iii) the difference between ventral left inferior frontal gyrus (vLIFG) and dorsal LIFG (dLIFG) connectivity (blue, reproduced from Fig. 6). In the main figure, the top row displays the Neurosynth functional connectivity pattern for a seed corresponding to the centre of gravity (COG) for the cluster where all three contrasts overlap. The bottom row compares this pattern for pMTG (in green) with the Noonan et al. (2013) semantic control meta-analysis from Fig. 1 (in red). Regions that fall within both maps are shown in yellow. Images are shown with fully saturated colours to maximise the visibility of the overlapping regions.

**Fig. 8:**
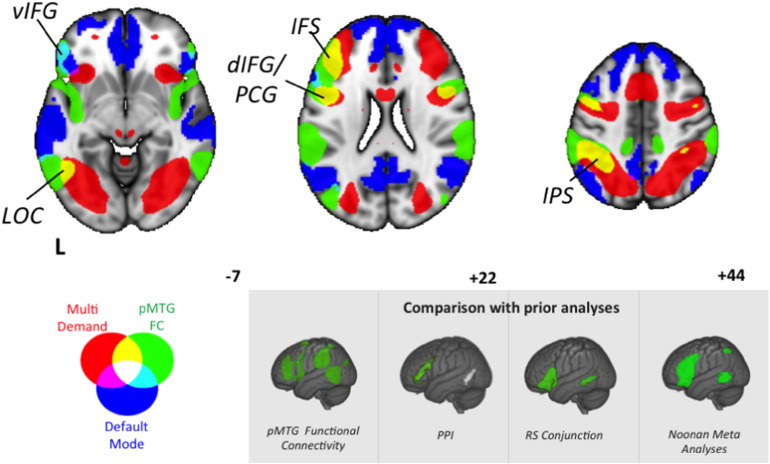
Functional connectivity of posterior middle temporal gyrus (pMTG; in green, reproduced from Fig. 7) contrasted with the multiple-demand network (MDN; in red) and default mode network (DMN; in blue). It can be seen that the connectivity of the pMTG intersects with the MDN and DMN in similar regions as the Noonan meta-analysis (see subpanel). Images are shown with fully saturated colours to maximise the visibility of the overlapping regions. vIFG = ventral inferior frontal gyrus; LOC = lateral occipital cortex; IFS = inferior frontal sulcus; dIFG/PCG = dorsal inferior frontal gyrus/precentral gyrus; IPS = intraparietal sulcus. For ease of visual comparison in the grey panel we show the spatial maps generated through our prior three analyses, as well the Noonan meta-analysis.

Our final analysis directly examined the similarity between the functional architecture of the pMTG region, identified by our analyses as important in event semantics, with the network associated with semantic control by the meta-analysis of Noonan et al. (2013). We calculated the conjunction of the previous three analyses (the task-based conjunction for action/relational semantic decisions; the resting-state connectivity conjunction for ATL and IFS; and the resting-state connectivity contrast of ventral IFG > dorsal IFG). This identified a common region centred on − 57 − 55 3 (Fig. 7 left panel). We entered this coordinate in Neurosynth (Yarkoni, 2011, Yarkoni et al., 2011) to examine its resting-state functional connectivity. The resulting map overlapped with the Noonan et al. (2013) meta-analysis in inferior frontal gyrus and also showed connectivity with temporal parietal junction and pre-SMA (Fig. 7 right panel). The connectivity map for pMTG was also compared with the DMN and the multiple-demand network (Fig. 8) and it extended into both of these networks, including overlap with the MDN in IFS, precentral gyrus (PCG), dorsal IFG, IPS and LOC, plus overlap with the DMN in ventral IFG and MTG.

## Discussion

This study set out to better characterise the functional role of the posterior middle temporal gyrus (pMTG) in semantic processing. Left pMTG is thought to play a key role in both controlled aspects of semantic retrieval and the comprehension of events, relations and actions — but the specific relationship between structure and function remains an open question. Although the field of semantic cognition is converging on a component process account, involving conceptual representations plus control mechanisms which can shape the pattern of retrieval to suit the task or context, the brain mechanisms that underpin this capacity remain poorly understood. Our analysis suggests that pMTG is a functional nexus drawing together two well-documented large-scale networks implicated in automatic semantic processing and executive control, and thus allowing more controlled patterns of retrieval. In task-based fMRI, we identified pMTG through the conjunction of judgements about global semantic associations and action features and found that under these conditions it showed greater functional coupling with inferior regions of the left frontal prefrontal cortex that included aspects of both the DMN and the MDN. This region overlapped with an area implicated in semantic control by a recent meta-analysis (Noonan et al., 2013). Resting state functional connectivity in an independent data set revealed that the same region was intrinsically coupled at rest to seed regions exhibiting peak activity in hard > easy semantic judgements (in inferior frontal sulcus, IFS, within the multiple-demand executive network) and easy > hard decisions (in anterior temporal lobe; ATL). A similar analysis using diffusion MRI data demonstrated that long-range connections from IFS and ATL overlapped in white matter adjacent to pMTG. Together these findings show that in topological terms, pMTG is located at the intersection of the DMN and MDN, a position that would allow it to integrate information from otherwise anti-correlated large-scale systems.

Our findings also link the functions of pMTG to previously-reported dissociations between different aspects of semantic control within left inferior frontal gyrus (LIFG; Badre et al., 2005). Anterior ventral LIFG is implicated in ‘controlled retrieval’, while posterior dorsal LIFG is involved in the ‘selection’ of relevant information (Badre et al., 2005). Our study suggests that this distinction extends to the posterior temporal cortex: pMTG was differentially linked to ventral IFG, consistent with previous work which identified strong connectivity between these regions for semantically demanding sentences (Snijders et al., 2010). In contrast, posterior inferior temporal gyrus (ITG) and lateral occipital cortex (LOC; below but adjacent to pMTG) were coupled with IFS and implicated in demanding feature selection tasks. Our ROI analysis of the task-based data revealed that both ventral IFG (i.e., the site implicated in controlled retrieval by Badre et al., 2005) and pMTG (the site linked to semantic control by Noonan et al., 2013) showed a pattern of functional recruitment consistent with a role in event comprehension — i.e., a stronger response to action vs. colour judgments and to global vs. size judgements. In contrast, dorsal IFG showed a stronger response in the hardest feature matching trials, irrespective of the need to identify a spatiotemporal or thematic context: both colour and action features elicited more activation in this region than global semantic associations. pMTG also showed stronger connectivity with ventral than dorsal IFG in resting-state analyses. This pattern is consistent with the view that dorsal IFG bordering IFS is implicated in the application of a goal to semantic selection, while ventral IFG and pMTG allow retrieval to be shaped to suit a context defined by the stimuli themselves (i.e., link is not specified by the task instructions). Global associations, especially those tapping relatively weak, non-dominant relationships (which elicit more activation than strong associations in pMTG across multiple studies; Badre et al., 2005, Gold et al., 2005, Noonan et al., 2013, Krieger-Redwood et al., 2015), require retrieval mechanisms to determine a context which links these items together and applied to ensure retrieval stays focussed on the association being probed. Action feature matching also requires a spatiotemporal context to be determined and used to guide the retrieval of actions. Finally, we used the conjunction of the location of the pMTG from each of these analyses as a seed region in a publicly available data set, yielding a pattern of connectivity that was consistent with a meta-analysis of studies of semantic control (Noonan et al., 2013).

Together these lines of structural and functional evidence constrain the possible role of pMTG in semantic cognition. The spatial correspondence between event semantics and a meta-analysis of semantic control rules out simple interpretations of this region as supporting only one or other of these aspects of processing. Likewise, our demonstration that pMTG is a convergence zone for both the DMN and the MDN, while occupying a spatial location that is independent of regions recruited by easy and hard decisions, indicates that this region is not an exclusive member of either network. Our PPI analysis shows that during event semantics the pMTG becomes functionally coupled to both regions of the DMN and the MDN, providing evidence that the pMTG is in communication with multiple networks when semantic retrieval must be shaped to fit different contexts. Contextual shaping of retrieval is common to both event semantics and semantic control, and could be made possible by the location of the pMTG as a nexus between the DMN and the MDN. Since unguided spreading activation in ATL recovers strong associations and features, even when these are irrelevant, this process may lead to problems in retrieval when the appropriate target is not the most dominant aspect of our knowledge. By integrating information from the DMN and MDN the pMTG may allow a set of features to be maintained that can help *shape* ongoing spreading activation to suit a particular spatiotemporal or thematic context, as occurs in both weak associations, and tasks that depend upon spatiotemporal context such as event semantics. Our analysis highlights that pMTG is spatially well-placed to provide this constraint on semantic retrieval since it is located between systems that are important for automatic semantic retrieval and top-down goal-directed cognition.

Our study is also informative regarding the functional similarities and differences between angular gyrus (AG) and pMTG. Consistent with the hypothesis that AG/pMTG form a convergence zone for thematic knowledge, both of these sites have been implicated in event semantics and thematic associations, as opposed to object knowledge (Schwartz et al., 2011). Nevertheless, pMTG has previously been implicated alongside LIFG in semantic control, while AG tends to show task-related deactivation along with aspects of ATL, especially for non-semantic tasks and harder semantic tasks (Humphreys et al., 2015). While AG showed a response to easy global associations over hard feature selection in this study, and strong resting state connectivity to ATL, pMTG did not show the same profile — the response of this region was not explicable in terms of overall difficulty (or reverse difficulty) and it showed connectivity at rest to both ATL and IFS. Our data, therefore, add to a growing body of evidence that while AG and pMTG sometimes co-activate during semantic tasks, they do not always do so, and this may be in part a consequence of their differential patterns of functional connectivity.

In summary, the current study helps constrain component process accounts of semantic cognition. There is an emerging consensus that the brain regions reliably activated by semantic tasks can show different response profiles — for example, meta-analytic investigations of neuroimaging evidence, work with patient populations and TMS studies in normal participants suggest that brain regions implicated in automatic semantic retrieval and representation can be dissociated from others implicated in more controlled semantic processing (Jefferies and Lambon Ralph, 2006, Jefferies, 2013, Noonan et al., 2013, Humphreys and Lambon Ralph, 2014). In addition, only a subset of the regions implicated in semantic control contributes to executively-demanding *non-semantic* judgements (Noonan et al., 2013). In line with this literature, our data provide converging evidence for at least three components of semantic cognition. First, a network engaging ATL, AG, medial PFC and PCC, broadly corresponding to the so-called default mode network, responds to more strongly to relatively easy global associations, suggesting that it preferentially supports automatic spreading activation within semantic representations. Second, a network including IFS, dorsal posterior IFG, IPS, pre-SMA and ITG/LOC, corresponding to the multiple-demand executive network, is recruited for demanding feature decisions suggesting that it might be important for the top down allocation of effort to match current environmental demands. Finally, pMTG and aIFG formed a third network showing common recruitment in situations when semantic retrieval must be shaped in a flexible way to suit a spatiotemporal or thematic context (even for global associations; i.e., in the absence of task instructions that require goal-driven retrieval). Thus, we propose that pMTG facilitates integration of information from regions in the DMN (that underpin more automatic aspects of semantic cognition) and those in the MDN (that contribute to executively-demanding judgements), together with more ventral aspects of IFG. This third component of semantic cognition relies on the integration of DMN and MDN that would otherwise remain anti-correlated, allowing the retrieval of semantic representations to be shaped to fit the current demands.

## Figures and Tables

**Table 1:** Psycholinguistic variables for the stimuli used in the task-based fMRI study.

Condition	Word length		Number of words		Manipulability		Lexical frequency		Familiarity		Imageability	
Mean	SD	Mean	SD	Mean	SD	Mean	SD	Mean	SD	Mean	SD
Global association	6.92	1.71	1.23	.26	4.77	.61	3.98	.39	5.74	.57	5.96	.51
Size feature	7.45	1.52	1.24	.27	4.77	.53	3.89	.43	5.88	.55	6.09	.42
Specific feature: action	7.13	1.64	1.27	.37	4.35	.44	3.94	.06	5.93	.46	6.30	.24
Specific feature: colour	7.63	1.87	1.28	.22	5.29	.33	3.81	.37	5.75	.49	5.67	.39

**Table 2:** Behavioural results (RT, accuracy, and response efficiency).

Condition	Response efficiency		RT		Accuracy	
Mean	SE	Mean (milliseconds)	SE	Mean (% correct)	SE
Animal global	1636.8	59.9	1565.1	52.5	.96	.01
Tool global	1984.2	94.2	1594.7	62.7	.97	.01
Animal size	2292.7	154.3	1830.5	52.2	.94	.01
Tool size	1674.9	84.5	1896.7	45.8	.90	.02
Animal colour	2192.8	112.2	1866.9	50.1	.86	.03
Tool action	2333.4	118.5	2031.7	61.1	.90	.02

SE = standard error.

## Appendix A Supplementary data

Supplementary tables. Image 2
