# Supplementary material for: Exploring the role of the posterior middle temporal gyrus in semantic cognition: Integration of anterior temporal lobe with executive processes
Source: Neuroimage. 2016 Aug 15;137:165–77. doi: 10.1016/j.neuroimage.2016.05.051 (PMC4927261; doi:10.1016/j.neuroimage.2016.05.051)
Supplement: Supplementary file 1 — Supplementary tables. [file mmc1.pdf]

Table S1. Cluster COG, size, and sub-peaks for the contrasts and conjunction in the top row of Figure 3. Peaks were identified in z-statistic spatial maps following cluster correction for multiple comparisons with a Z inclusion threshold of 2.3 and a cluster significance threshold set at p <.05.

	Cluster size (Voxels)	Cluster p-value (-log10)	Z	MNI coordinates X	Y	Z	Hemisphere	Location
**Action > colour**								
Cluster 1 COG (-44 -53 26)	7814	14	5.25	-58	-66	-6	LH	Inferior Lateral Occipital cortex
			5.22	-58	-68	-12	LH	Inferior Lateral Occipital cortex
			5.17	-52	-44	36	LH	Posterior Supramarginal gyrus
			5.12	-60	-34	30	LH	Anterior Supramarginal gyrus
			5.08	-54	-32	44	LH	Anterior Supramarginal gyrus
			5.04	-38	-54	52	LH	Superior Parietal Lobule
Cluster 2 COG (-27 -4 50)	2416	5.48	4.62	-36	-6	46	LH	Precentral gyrus
			3.91	-24	-14	46	LH	Precentral gyrus
			3.91	-22	-10	42	LH	Left cerebral white matter
			3.88	-28	-10	48	LH	Precentral gyrus
			3.87	-22	0	46	LH	Superior Frontal gyrus
			3.85	-36	-8	36	LH	Left cerebral white matter
Cluster 3 COG (-48 20 10)	2022	4.67	5.27	-52	10	6	LH	Inferior Frontal gyrus, pars opercularis
			4.27	-48	32	-4	LH	Inferior Frontal gyrus, pars opercularis
			4.19	-42	30	-2	LH	Frontal Orbital cortex
			4.08	-52	4	20	LH	Precentral gyrus
			4.05	-50	24	12	LH	Inferior Frontal gyrus, pars triangularis
			3.91	-52	40	-2	LH	Frontal pole
**Global all > size all**								
Cluster 1 COG (-51 -32-2)	6967	11.2	5.01	-58	-32	-8	LH	Posterior Middle Temporal gyrus
			5.01	-44	-62	26	LH	Superior Lateral occipital cortex
			4.99	-42	-68	32	LH	Superior Lateral occipital cortex
			4.88	-68	-44	-8	LH	Posterior Middle Temporal gyrus
			4.84	-60	-56	2	LH	Temporooccipital Middle Temporal gyrus
			4.76	-54	-56	30	LH	Angular gyrus
Cluster 2 COG (-52 -35-1)	6485	10.6	5.15	58	-52	4	RH	Superior middle temporal gyrus
			5.04	60	-54	16	RH	Angular gyrus
			5.01	58	-44	0	RH	Temporooccipital Middle Temporal gyrus
			5.01	60	-48	2	RH	Temporooccipital Middle Temporal gyrus
			4.49	46	-82	10	RH	Inferior Lateral Occipital cortex
			4.42	54	-10	-22	RH	Posterior Middle Temporal gyrus
Cluster 3 COG (-7 39 31)	6301	10.3	4.71	-16	38	44	LH	Superior Frontal gyrus
			4.65	-16	56	30	LH	Frontal pole
			4.22	-42	10	52	LH	Middle Frontal gyrus
			4.06	-42	6	52	LH	Middle Frontal gyrus
			4.06	-40	2	52	LH	Middle Frontal gyrus
			3.94	-8	50	-6	LH	Paracingulate gyrus
Cluster 4 COG (1 -57 35)	3273	6.11	5.28	-6	-60	28	LH	Precuneous cortex
			4.83	8	-54	30	RH	Precuneous cortex
			4.62	-2	-50	28	LH	Cingulate gyrus
			3.73	2	-54	44	RH	Precuneous cortex
			3.46	8	-52	50	RH	Precuneous cortex
			3.43	-8	-48	50	LH	Precuneous cortex
Cluster 4 COG (12 -81 -25)	2872	4.49	4.49	16	-86	-36	RH	Cerebellum
			4.3	32	-80	-42	RH	Cerebellum
			4.26	18	-86	-42	RH	Cerebellum

			4.22	46	-80	-12	RH	Inferior Lateral Occipital cortex
			4.2	30	-88	-30	RH	Inferior Lateral Occipital cortex
			3.83	20	-78	-34	RH	Cerebellum
**Action > colour & global all > size all conjunction**								
Cluster 1 COG (-50-60 14)	689	1.42	4.45	-58	-56	0	LH	Temporooccipital Middle Temporal gyrus
			4.21	-56	-54	-4	LH	Temporooccipital Middle Temporal gyrus
			3.62	-54	-52	28	LH	Angular gyrus
			3.32	-46	-60	4	LH	Temporooccipital Middle Temporal gyrus
			3.23	-42	-62	8	LH	Inferior Lateral Occipital cortex
			3.21	-38	-60	10	LH	Temporooccipital Middle Temporal gyrus

Table provides data for all significant clusters and sub-peaks within each cluster, with the first sub-peak being the cluster maxima. Location was determined using the Harvard-Oxford cortical structural atlas built into FSL. COG = centre of gravity.

Table S2. Cluster COG, size, and sub-peaks for the contrasts and conjunction in the bottom row of Figure 3. Peaks were identified in z-statistic spatial maps following cluster correction for multiple comparisons with a Z inclusion threshold of 2.3 and a cluster significance threshold set at p <.05.

	Cluster size (Voxels)	Cluster p-value (-log10)	MNI coordinates				Hemisphere	Location
			Z	X	Y	Z		
**Feature selection > global semantics**								
Cluster 1 COG (-38 15 10)	4760	8.93	5.8	-42	28	16	LH	Inferior Frontal gyrus, pars triangularis
			5.68	-46	38	6	LH	Frontal pole
			5.55	-46	42	0	LH	Frontal pole
			5.34	-32	18	-4	LH	Insular cortex
			5.33	-30	22	-6	LH	Insular cortex
			5.27	-40	0	28	LH	Precentral gyrus
Cluster 2 COG (-40 -49 40)	2542	5.3	5.4	-40	-50	40	LH	Angular gyrus
			5.02	-28	-54	30	LH	Superior Lateral Occipital cortex
			4.99	-28	-72	42	LH	Superior Lateral Occipital cortex
			4.29	-50	-38	42	LH	Anterior Supramarginal gyrus
			4.03	-44	-40	28	LH	Parietal Operculum cortex
			3.71	-36	-66	56	LH	Superior Lateral Occipital cortex
Cluster 3 COG (-36 -60 -17)	1258	2.7	5.04	-42	-58	-10	LH	Temporooccipital Inferior Temporal gyrus
			4.18	-50	-60	-18	LH	Temporooccipital Inferior Temporal gyrus
			4.09	10	-80	-32	RH	Cerebellum
			3.25	-24	-48	-24	LH	Cerebellum
			3.11	-8	-80	-34	LH	Cerebellum
			3.09	-36	-46	-22	LH	Temporal Occpital Fusiform cortex
Cluster 4 COG (-3 18 44)	1103	2.09	6.02	-6	20	40	LH	Paracingulate gyrus
			3.8	6	24	44	RH	Paracingulate gyrus
			3.73	10	22	42	RH	Paracingulate gyrus
			3.06	8	30	36	RH	Paracingulate gyrus
			2.76	-10	4	56	LH	Justapositional Lobule cortex
Cluster 5 COG (46 24 17)	882	1.79	5.29	42	34	14	RH	Inferior Frontal gyrus, pars triangularis
			4.49	46	6	22	RH	Precentral gyrus
			3.78	54	38	10	RH	Frontal pole
			3.53	36	6	22	RH	Precentral gyrus
			3.19	46	22	18	RH	Inferior Frontal gyrus, pars triangularis
			2.95	56	20	28	RH	Inferior Frontal gyrus, pars opercularis
**Global semantics > feature selection**								
Cluster 1 COG (19 -52 27)	17163	23.2	6.11	-6	-60	24	LH	Precuneus cortex
			5.87	-6	-52	24	LH	Posterior Cingulate gyrus
			5.77	-2	-68	22	LH	Precuneus cortex
			5.68	10	-60	26	RH	Precuneus cortex
			5.67	56	-10	-22	RH	Posterior Middle Temporal gyrus
			5.63	2	-54	50	RH	Precuneus cortex
Cluster 2 COG (2 45	7451	12.6	5.16	0	52	-10	RH	Frontal medial cortex
			5.04	8	56	0	RH	Frontal pole

19)			4	0	56	2	RH	Frontal pole
			4.87	-4	40	6	LH	Cingulate gyrus
			4.82	-14	50	38	LH	Frontal pole
			4.71	14	52	30	RH	Frontal pole
Cluster 3 COG (-48 68 26)	1438	3.1	5	-40	-78	34	LH	Superior Lateral Occipital cortex
			4.5	-52	-68	22	LH	Superior Lateral Occipital cortex
			4.42	-56	-66	16	LH	Superior Lateral Occipital cortex
			4.41	-46	-78	26	LH	Superior Lateral Occipital cortex
			4.29	-52	-68	28	LH	Superior Lateral Occipital cortex
			4.19	-44	-60	26	LH	Angular gyrus
Cluster 4 COG (-58 -17 -18)	1074	2.26	5.33	-64	-28	-8	LH	Posterior Middle Temporal gyrus
			5.31	-62	-20	-12	LH	Posterior Middle Temporal gyrus
			4.68	-56	-14	-22	LH	Posterior Middle Temporal gyrus
			4.32	-48	2	-38	LH	Temporal pole
			3.92	-56	-6	-28	LH	Anterior Middle Temporal gyrus
			2.93	-52	6	-28	LH	Temporal pole

Table provides data for all significant clusters and sub-peaks within each cluster, with the first sub-peak being the cluster maxima. Location was determined using the Harvard-Oxford cortical structural atlas built into FSL. COG = centre of gravity.

Table S3. Cluster COG, size, and sub-peaks for the PPI analysis in figure 4. Peaks were identified in z-statistic spatial maps following cluster correction for multiple comparisons with a Z inclusion threshold of 2.3 and a cluster significance threshold set at p <.05.

	Cluster size (Voxels)	Cluster p-value (-log10)	Z	MNI coordinates X	Y	Z	Hemisphere	Location
**Action > colour PPI**								
Cluster 1 COG (-10 -82 -11)	4729	19.5	5.01	-20	-98	-16	LH	Occipital pole
			4.77	-52	-60	-16	LH	Temporooccipital inferior temporal gyrus
			4.51	22	-94	-14	RH	Occipital pole
			4.45	36	-92	-12	RH	Inferior lateral occipital cortex
			4.19	-32	-90	-16	LH	Inferior lateral occipital cortex
			4.19	-48	-62	-8	LH	Inferior lateral occipital cortex/Temporooccipital inferior temporal gyrus
Cluster 2 COG (-44 20 19)	3422	15.3	5.03	-54	18	28	LH	Inferior frontal gyrus (pars opercularis)
			4.8	-54	12	30	LH	Inferior frontal gyrus (pars opercularis)/Precentral gyrus
			4.78	-46	44	-4	LH	Frontal pole
			4.69	-50	40	8	LH	Frontal pole
			4.59	-44	30	10	LH	Inferior frontal gyrus (pars triangularis)
			4.59	-46	24	18	LH	Inferior frontal gyrus (pars triangularis)
Cluster 3 COG (-24 -80 26)	1761	9.09	4.44	-24	-80	46	LH	Superior Lateral occpital cortex
			4.36	-24	-72	46	LH	Superior Lateral occpital cortex
			4.05	-36	-56	46	LH	Superior parietal lobule/Angular gyrus
			3.87	-32	-52	44	LH	Superior parietal lobule/Posterior Supramarginal gyrus
			3.46	-52	-36	44	LH	Anterior Supramarginal gyrus
			3.37	-34	-60	52	LH	Superior Lateral occpital cortex
Cluster 4 COG (-4 18 32)	453	2.41	5.12	-4	18	42	LH	Paracingulate gyrus
**Global > size PPI**								
Cluster 1 COG (-49 23 14)	1612	8.45	4.61	-52	24	18	LH	Inferior frontal gyrus (pars triangularis)
			4.55	-56	24	20	LH	Inferior frontal gyrus (pars triangularis)
			4.4	-54	32	10	LH	Inferior frontal gyrus (pars triangularis)
			4.39	-46	18	20	LH	Inferior frontal gyrus (pars opercularis)
			3.9	-38	6	22	LH	Inferior frontal gyrus (pars opercularis)
			3.86	-52	38	0	LH	Frontal pole
**Action > colour PPI & Global > size PPI conjunction**								
Cluster 1 COG (-48 23 15)	1262	6.92	4.26	-50	24	18	LH	Inferior frontal gyrus (pars triangularis)
			3.94	-38	14	20	LH	Inferior frontal gyrus (pars opercularis)
			3.9	-38	6	22	LH	Inferior frontal gyrus (pars opercularis)
			3.86	-52	38	0	LH	Frontal pole
			3.81	-48	30	14	LH	Inferior frontal gyrus (pars triangularis)
			3.58	-52	36	8	LH	Inferior frontal gyrus (pars triangularis)

Table provides data for all significant clusters and sub-peaks within each cluster, with the first sub-peak being the cluster maxima. Location was determined using the Harvard-Oxford cortical structural atlas built into FSL. COG = centre of gravity.

Table S4. Cluster COG, size, and sub-peaks for the resting-state fMRI networks in figure 5. Peaks were identified in z-statistic spatial maps following cluster correction for multiple comparisons with a Z inclusion threshold of 2.3 and a cluster significance threshold set at p <.05.

	Cluster size (Voxels)	Cluster p-value (-log10)	Z	MNI coordinates X	Y	Z	Hemisphere	Location
**-45 39 3**								
Cluster 1 COG (-41 0 10)	18706	26.4	21.9	-44	38	2	LH	Frontal pole/Inferior Frontal gyrus, pars triangularis
			12.1	-46	8	20	LH	Inferior Frontal gyrus, pars opercularis/Precentral gyrus
			11.8	-52	10	14	LH	Inferior Frontal gyrus, pars opercularis
			11.7	-50	18	22	LH	Inferior Frontal gyrus, pars opercularis
			10.4	-56	-60	-8	LH	Temporooccipital middle temporal gyrus
			9.22	-28	18	-2	LH	Insular cortex
Cluster 2 COG (48 42 0)	5085	12.6	12.6	48	42	0	RH	Frontal pole
			8.06	26	34	-14	RH	Frontal pole/Frontal orbital cortex
			7.64	56	16	16	RH	Inferior Frontal gyrus, pars opercularis
			6.68	46	10	28	RH	Precentral gyrus/Inferior Frontal gyrus, pars opercularis
			5.42	40	28	6	RH	Inferior Frontal gyrus, pars triangularis
			5.41	42	32	-4	RH	Frontal orbital cortex/Inferior Frontal gyrus, pars triangularis
Cluster 3 COG (24 -74 -35)	1776	8.27	8.27	12	-78	-32	RH	Cerebellum
			7.33	30	-68	-34	RH	Cerebellum
			7.08	34	-68	-44	RH	Cerebellum
			5.27	20	-76	-44	RH	Cerebellum
Cluster 4 COG (58 -50 -11)	678	6.05	6.05	56	-48	-10	RH	Temporooccipital Inferior/middle Temporal gyrus
			2.54	52	-48	-24	RH	Temporooccipital inferior temporal gyrus
			2.48	50	-46	-30	RH	Temporooccipital inferior temporal gyrus
**-58 -49 -9**								
Cluster 1 COG (-24 -31 1)	23986	30.6	22.4	-60	-50	-10	LH	Temporooccipital middle temporal gyrus
			12.3	60	-46	-10	RH	Temporooccipital middle temporal gyrus
			11.9	-42	46	-12	LH	Frontal pole
			10.9	-50	36	10	LH	Inferior Frontal gyrus, pars triangularis
			10.9	32	-72	-44	RH	Cerebellum
			10.6	-34	-74	44	LH	Superior lateral occipital cortex
Cluster 2 COG (48 42 0)	1965	4.66	6.86	38	40	-14	RH	Frontal pole
			5.76	44	52	-12	RH	Frontal pole
			5.53	46	16	32	RH	Middle frontal gyrus
			5.38	48	28	20	RH	Inferior Frontal gyrus, pars triangularis
			5.06	50	38	10	RH	Frontal pole/Inferior Frontal gyrus, pars triangularis
			4.39	38	12	32	RH	Middle frontal gyrus
Cluster 3 COG (42 -62 43)	1341	3.31	6.49	36	-66	48	RH	Superior lateral occipital cortex
			5.81	40	-64	38	RH	Superior lateral occipital cortex
			4.35	48	-56	50	RH	Angular gyrus
			3.88	52	-48	50	RH	Angular gyrus/Supramarginal gyrus
**Conjunction**								
Cluster 1	2709	5.58	7.17	-40	30	-14	LH	Frontal orbital cortex

COG (-42 21 -8)			7.15	-44	32	-12	LH	Frontal orbital cortex
			7.12	-50	28	-4	LH	Inferior Frontal gyrus, pars triangularis
			6.97	-56	22	8	LH	Inferior Frontal gyrus, pars triangularis
			6.94	-54	24	4	LH	Inferior Frontal gyrus, pars triangularis
			6.88	-52	18	10	LH	Inferior Frontal gyrus, pars opercularis
			5.81	-62	-44	-10	LH	Posterior middle temporal gyrus
			5.07	-30	0	-42	LH	Anterior temporal fusiform cortex
Cluster 2 COG (-46 -37 -17)	1092	2.34	5.02	-40	-38	-22	LH	Posterior temporal fusiform cortex
			4.51	-42	-44	-26	LH	Posterior temporal fusiform cortex
			4.31	-44	-48	-30	LH	Temporal occpital fusiform cortex
			3.91	-34	-12	-40	LH	Posterior temporal fusiform cortex
			6.88	-8	30	48	LH	Superior frontal gyrus
			5.97	-10	26	50	LH	Superior frontal gyrus
Cluster 3 COG (-10 38 37)	762	1.6	5.48	-8	18	52	LH	Superior frontal gyrus
			3.98	-14	60	18	LH	Frontal pole
			3.96	-16	56	18	LH	Frontal pole
			3.08	-8	40	20	LH	Paracingulate gyrus/anterior cingulate

Table provides data for all significant clusters and sub-peaks within each cluster, with the first sub-peak being the cluster maxima. Location was determined using the Harvard-Oxford cortical structural atlas built into FSL. COG = centre of gravity.

Table S5. Cluster COG, size, and sub-peaks for the resting-state fMRI networks in figure 6. Peaks were identified in z-statistic spatial maps following cluster correction for multiple comparisons with a Z inclusion threshold of 2.3 and a cluster significance threshold set at p <.05.

	Cluster size (Voxels)	Cluster p-value (-log10)	Z	MNI coordinates X	Y	Z	Hemisphere	Location
**-48 18 18 > -51 27 -3**								
Cluster 1 COG (-40 -3 17)	18363	25.8	22.4	-50	18	20	LH	Inferior Frontal gyrus, pars opercularis
			11.8	-44	6	40	LH	Middle frontal gyrus
			11.8	-40	4	48	LH	Middle frontal gyrus
			11.8	-46	44	-6	LH	Frontal pole
			11	-4	34	44	LH	Superior frontal gyrus/paracingulate gyrus
			10.4	-32	12	50	LH	Middle frontal gyrus
Cluster 2 COG (50 13 13)	5155	10.3	13	48	24	20	RH	Inferior Frontal gyrus, pars triangularis/opercularis
			6.49	46	6	46	RH	Middle frontal gyrus/precentral gyrus
			5.78	52	36	-10	RH	Frontal pole
			5.06	60	-42	-6	RH	Temporooccipital middle Temporal gyrus
			5	68	-38	-6	RH	Temporooccipital middle Temporal gyrus
			4.83	34	24	-6	RH	Frontal orbital cortex
Cluster 3 COG (24 -75 -34)	2099	5.02	8.92	14	-76	-32	RH	Cerebellum
			7.85	28	-70	-44	RH	Cerebellum
			7.79	32	-70	-34	RH	Cerebellum
**-51 27 -3 > -48 18 18**								
Cluster 1 COG (-37 6 9)	21198	28.8	22	-52	26	-4	LH	Inferior Frontal gyrus, pars triangularis
			12.6	-6	16	56	LH	Superior frontal gyrus
			11.4	-54	-38	-6	LH	Posterior middle temporal gyrus
			10.9	-6	30	50	LH	Superior frontal gyrus
			10.7	-60	-50	28	LH	Supramarginal gyrus/Angular gyrus
			10.6	-8	46	40	LH	Frontal pole/Superior frontal gyrus
Cluster 2 COG (47 11 -1)	6520	12.4	13.2	50	30	-6	RH	Frontal orbital cortex/Inferior Frontal gyrus, pars opercularis
			9.54	36	26	-16	RH	Frontal orbital cortex
			8.4	54	-34	-2	RH	Posterior middle temporal gyrus
			5.99	16	10	8	RH	Right caudate
			5.87	54	12	-22	RH	Temporal pole
			5.4	36	12	-4	RH	Insular cortex
Cluster 3 COG (26 -76 -34)	2155	5.11	9.61	26	-78	-36	RH	Cerebellum
			9.47	24	-78	-32	RH	Cerebellum
			4.36	48	-62	-34	RH	Cerebellum

Table provides data for all significant clusters and sub-peaks within each cluster, with the first sub-peak being the cluster maxima. Location was determined using the Harvard-Oxford cortical structural atlas built into FSL. COG = centre of gravity.
